# Academic pressure and academic procrastination: The mediating role of negative coping strategies

**DOI:** 10.1371/journal.pone.0338956

**Published:** 2025-12-19

**Authors:** Chuanjia Xue, Zhiwei Helian, Yue Li

**Affiliations:** 1 College of Economics and Management, Yanshan University, Qinhuangdao, China; 2 Hebei University of Traditional Chinese Medicine, Shijiazhuang, China; University of Central Punjab, PAKISTAN

## Abstract

Rooted in the Extended Procrastination-Health Framework, this study employed a survey of 600 Chinese university students to elucidate the mechanistic relationships among academic pressure, coping strategies, and academic procrastination. The findings reveal: (1) Academic pressure was positively associated with the increased academic procrastination (β = 0.470, p < 0.001); (2) Academic pressure was significantly positively associated with increased negative coping styles (β = 0.426, p < 0.001), while the negative coping styles was positively associated with the increased academic procrastination (β = 0.158, p < 0.05). (3) Structural equation modeling confirmed that negative coping strategies partially mediate the academic pressure-procrastination nexus (indirect effect = 0.068, proportionate contribution 12.64%), with the model demonstrating excellent fit indices (χ²/df = 2.89, CFI = 0.93, RMSEA = 0.05). Notably, positive coping strategies exhibited no significant mediating effect. This study validates the theoretical pathway “academic pressure - negative coping - academic procrastination” and underscores the important bridging function of negative coping strategies in translating stress into procrastinatory behavior. The results provide empirically grounded intervention targets for universities to disrupt procrastination cycles through targeted emotional regulation and stress management programs.

## 1. Introduction

In contemporary higher education systems, the intensification of academic pressure and the pervasive nature of academic procrastination have emerged as salient challenges impacting university students’ learning efficacy and psychosocial well-being. The reason of academic pressure is multifactorial, encompassing personal, social, familial, and institutional stressors [1–7]. Academic procrastination is conceptually defined as the voluntary delay of academically relevant tasks despite cognitive awareness of the need for timely completion, which usually manifest by behavior as task avoidance, cognitive distraction, and engagement in non-academic digital activities [] [8]. Meta-analytic evidence indicates that approximately 50% of university students demonstrate clinically significant levels of academic procrastination [1,2], with this behavior exhibiting a strong positive correlation with anxiety symptoms (r = 0.38, p < 0.001) [9]. While acute and low-intensity stressors have been shown to enhance academic performance and physiological resilience [10], chronic and high-intensity stressors – when coupled with inadequate coping resources – predict diminished academic achievement and compromised mental health outcomes [11–18]. Longitudinal studies further demonstrate that high-pressure academic environments exacerbate procrastinatory tendencies, which reciprocally amplify perceived stress, creating a self-reinforcing vicious cycle [9].

Despite extensive prior research examining correlates of academic procrastination – including perfectionistic traits, achievement goal orientations, future time perspective, and chronotype preferences [19]—the process-oriented mechanisms through which coping strategies mediate the pressure-procrastination relationship remain underexplored, particularly within Chinese university contexts. Existing literatures predominantly employ cross-sectional designs to describe procrastination prevalence or isolate univariate effects, lacking rigorous process-oriented frameworks that delineate how distinct coping strategies dynamically influence stress-procrastination pathways. This study addresses these gaps by adopting a structural equation modeling approach to examine the mediating role of coping strategies in the academic pressure-procrastination relationship, with specific attention to behavioral response patterns among Chinese university students in high-competition academic environments. The study contributes theoretically in extending the Procrastination-Health Model through empirical validation of the negative coping pathway, while provides practical implications include evidence-based guidelines for psychological interventions in higher education institutions.

## 2. Theoretical background and conceptual framework

### 2.1. Concept and impact of academic procrastination

Academic procrastination is defined as the voluntary delay in initiating or completing prescribed learning tasks despite awareness of potential negative consequences, representing a prototypical manifestation of self-regulation failure [20,21]. Three core dimensions have been empirically delineated: irrational delay, concomitant subjective distress, and substantive impairment to academic efficacy [22]. The advent of digital technologies has given rise to “digital procrastination” as a novel behavioral manifestation, characterized by frequent interruptions of study processes for online activities [23].

Primary antecedents of academic procrastination are identified as fear of failure and task aversion [24]. According to self-regulation theory, when confronted with aversive tasks, emotional regulation is prioritized over cognitive regulation, with students favoring immediate emotional relief strategies (e.g., entertainment) over goal-directed problem-solving [18]. Recent theoretical advances integrating temporal decision-making frameworks further suggest that procrastination fundamentally stems from hypersensitivity to task-related aversive valence and discounting of delayed rewards, leading to perpetual deferral of tasks to “future selves” [25]. Pressure-coping theory additionally indicates that when academic demands are appraised as threatening, students tend to adopt avoidance coping strategies, thereby initiating procrastination cycles [26].

The consequences of academic procrastination are multidimensional: meta-analytic evidence demonstrates moderate correlations with GPA decline and increased academic failure risk; longitudinal studies confirm its exacerbation of anxiety, depressive symptoms, and reduced subjective well-being [27]. Notably, a bidirectional relationship is observed between procrastination and sleep disturbances, where nocturnal procrastination compresses restorative sleep duration, subsequently weakening next-day self-regulatory capacity [28]. Chronic procrastination solidifies maladaptive behavioral patterns that further impair academic performance through self-efficacy erosion [26]. Thus, academic procrastination is recognized not only as a critical determinant of academic achievement but also as a pivotal issue affecting university students’ psychological well-being and behavioral development, necessitating systematic intervention.

### 2.2. Academic pressure and academic procrastination

Academic pressure is characterized as the psychological load and tension experienced by university students in academic contexts, relating to learning objectives, achievement expectations, and educational resource availability, with stress responses arising when individuals perceive resource inadequacy to meet academic demands [29]. Contemporary research has systematized its multidimensional structure into four primary components: academic task pressure, competitive pressure, self-expectation pressure, and environmental pressure [30]. Within Chinese educational contexts, collectivist cultural values amplify “achievement-oriented” social comparison pressures, resulting in high-intensity, persistent academic stress among Chinese university students [31].

Dual effects of academic pressure are evident to the development of students: mild stress has been shown to have a positive impact on students’ learning motivation, whereas chronic excessive pressure would induce negative mental consequences as anxiety, depression, sleep disorders [22], and academic burnout [32]. According to ego depletion theory sustained stress would exposure depletes regulatory resources, impairing inhibitory control over procrastinatory impulses; while high stress would further consume executive function resources, leading to attentional fragmentation and diminished self-regulation [33].

In some empirical studies, immediate hedonic activities are confirmed to have priorities over aversive task engagement to alleviate the negative affect of students [34], while stress-induced negative emotions amplify task-aversive appraisals, reinforcing procrastinatory behavior [35]. Recent longitudinal research also revealed the bidirectional pressure-procrastination dynamics: initial pressure predicts subsequent procrastination levels, and accumulated unfinished tasks exacerbate stress perceptions would form a vicious cycle [36]. The elucidation of these mechanisms holds significant theoretical and practical value for disrupting procrastination cycles.

### 2.3. Coping strategies and academic procrastination

Pressure coping strategies are defined as cognitive-behavioral responses to stress, categorized into positive (problem-focused) and negative (emotion-focused) dimensions [37]. Positive coping encompasses problem-solving, social support seeking, and cognitive reappraisal [27]; while negative coping includes avoidance, self-blame, and fantasy [38]. Recent validation studies confirm the framework’s robust applicability and predictive validity among Chinese university students [26].

Differential mechanisms of coping strategies are indicated by empirical evidence: negative coping correlates positively with academic procrastination through resource depletion and executive dysfunction, while positive coping may buffer stress-procrastination linkages via structured planning and resource augmentation through social support.

However, inconsistent findings regarding multiple moderating factors necessitate further investigation. The aim of this study is to clarify the differential mechanisms of coping strategies to inform targeted intervention designs for procrastination reduction.

### 2.4. Applicability of the extended procrastination-health model

An integrative framework for understanding the dynamic health impacts of procrastination is provided by Fuschia M. Sirois’s Extended Procrastination-Health Model. It is posited by the model that irrational delay directly generates task accumulation and time pressure, amplifying stress perceptions; elevated stress subsequently depletes self-regulatory resources, promoting maladaptive short-term emotional relief strategies and perpetuating “procrastination-stress-procrastination” cycles with detrimental health consequences [39,40]. While the model has been validated in general procrastination and chronic illness cohorts, its applicability to high-competition academic environments among Chinese students remains untested. Key empirical questions include whether heightened academic pressure reliably predicts negative coping, and whether such coping exacerbates procrastinatory behavior in this population.

## 3. Research hypotheses development

This study aims to investigate the relationship mechanisms among academic pressure, coping strategies, and academic procrastination among Chinese university students within a cultural context. Specifically, it examines the impact of personal, familial, institutional, and social academic pressures on academic procrastination, with coping strategies serving as mediating variables. Based on literature review, the following hypotheses are proposed:

Hypothesis 1: Academic pressure and academic procrastination exhibit positive correlation among university students.

Hypothesis 2: Academic procrastination and adaptive coping strategies demonstrate negative correlation.

Hypothesis 3: Academic procrastination and negative coping strategies show positive correlation.

Hypothesis 4: Academic pressure and adaptive coping strategies exhibit positive correlation.

Hypothesis 5: Academic pressure and negative coping strategies demonstrate positive correlation.

Hypothesis 6: Positive coping strategies mediate the relationship between academic pressure and academic procrastination.

Hypothesis 7: Negative coping strategies mediate the relationship between academic pressure and academic procrastination.

## 4. Methodology

### 4.1. Ethical statement

This study adheres to the ethical guidelines of the American Psychological Association. The research protocol has been approved by the Ethics Committee of Hebei University of Chinese Medicine (Approval Number: YXLL202501003). On January 13, 2025, the Ethics Committee of Hebei University of Chinese Medicine held a meeting to review this research. The review documents included the ethical review application form, research protocol, informed consent form, subject safety protection plan, and the resume of the principal investigator, etc. After discussion, the committee deemed the research design reasonable and in line with ethical principles, and agreed to proceed with the project. Voting results: 7 attendees, 7 voters, 7 in favor, 0 against, 0 for further study, and 0 for suspension or termination of the study. Validity period: January 15, 2025 to March 1, 2025.

### 4.2. Participants

A stratified random sampling method was employed to recruit 600 university students from five higher education institutions in Hebei Province, yielding 579 valid responses. Sample distribution contains comprehensive universities (120 students, 20.7%), engineering institutions (233 students, 40.3%), normal universities (108 students, 18.7%), and agricultural/forestry universities (118 students, 20.4%). Detailed distribution is presented in Table 1.

**Table 1:** Sample Distribution Across Five Universities. University Name.

	Location	Discipline Category	Valid Responses	Percentage
Hebei University	Baoding	Comprehensive	120	20.7%
Yanshan University	Qinhuangdao	Engineering-oriented	115	19.9%
Hebei Normal University	Shijiazhuang	Teacher Education	108	18.7%
Hebei Agricultural University	Baoding	Agriculture/Forestry	118	20.4%
North China Electric Power University(Baoding Campus)	Baoding	Engineering	118	20.4%
Total			579	100%

### 4.3. Measurement instruments

Based on mature scales from domestic and international research, this study integrated three validated instruments—Academic Procrastination Scale, Academic Pressure Scale, and Coping Strategies Scale—into a unified questionnaire through systematic literature review and pilot interviews. Responses were recorded on a 5-point Likert scale (1 = Strongly Disagree, 5 = Strongly Agree).

The Scale of Academic Pressure of University Student was adapted to measure perceived academic pressure. The scale contains 20 items organized into four dimensions: personal pressure sources, familial pressure sources, institutional pressure sources, and social pressure sources.

To assess procrastination behavior, a questionaire of Academic Procrastination Questionnaire among University Students was used, which comprising 19 items categorized into three dimensions: delayed initiation, inadequate planning, and poor completion.

A Simplified Coping Style Questionnaire was utilized to measure the Coping Strategies. The questionaire featured 20 items divided into adaptive coping (e.g., seeking social support, positive reframing) and maladaptive coping (e.g., avoidance, substance use) dimensions, with concrete behavioral examples embedded in item descriptions.

### 4.4. Analytical strategy

First of all, this paper conducted a series of reliability and validity tests on the questionnaire. Internal consistency reliability was assessed using Cronbach’s alpha coefficients to detect the consistency of repeated measurements on the same object, with values exceeding 0.7 being considered indicative of acceptance. The validity test is a test of the effectiveness of the questionnaire, reflecting the validity and rationality of the questionnaire design. The commonly used test indicators are KMO and Bartlett ‘s spherical test. KMO > 0.8 indicates good validity. Bartlett ‘s sphericity test is used to test the correlation between variables, which is a key step to judge whether the data is suitable for dimensionality reduction analysis. Generally, Bartlett ‘s sphericity test is carried out before factor analysis, with p value as the core criterion. If p < 0.05, it indicates that there is a significant correlation between variables, and the data is suitable for factor analysis.

Secondly, this paper conducted a Harman single factor test to explore the influence of common method bias. Method bias is recognized as systematic errors caused by data collection method which might exaggerate or obscure the true relationship between variables. Common method bias may arise from data collection procedures such as respondents completing all items in a single session (potentially introducing fatigue or response consistency biases). Harman single factor test is to test whether all items are dominated by a single factor through exploratory factor analysis (EFA). In the principal component analysis, if the variance interpretation rate of the first unrotated factor is less than 40%, the deviation is not significant.

In this paper, SPSS23 was used to perform independent sample t-test and Pearson product difference correlation analysis. The independent sample t-test is mainly used to compare whether significant differences exist in the mean of two independent samples. Prior to these tests, Levene’s test for equality of variances was performed. Depending on the outcome of Levene’s test (p > 0.05 indicating equal variances), the appropriate row of the t-test output (“Equal variances assumed” or “Equal variances not assumed”) was selected. A statistically significant difference between groups was inferred when the p-value associated with the t-test was less than 0.05.

Pearson product-moment correlation coefficients were computed to assess the strength and direction of linear relationships between continuous variables. The magnitude of the correlations was interpreted according to standard criteria: |r| > 0.8 denoted a strong correlation, 0.5 ≤ |r| < 0.8 a moderate correlation, 0.3 ≤ |r| < 0.5 a weak correlation, and |r| < 0.3 a negligible correlation. Correlations were considered statistically significant when the accompanying p-value was less than 0.05.

To investigate the relationships among academic pressure, coping strategies, and academic procrastination, stepwise regression analysis was performed. This method iteratively introduced or removed predictor variables to construct an optimal regression model, retaining only those variables that significantly contributed to the prediction of the dependent variable (p < 0.05). Stepwise regression analysis was performed using the ‘ forward selection ‘ stepwise method based on the AIC criterion, the significance level for the included variables was p < 0.05 and the exclusion was p > 0.10.

Finally, structural equation modeling was conducted using Mplus to test for mediation effects. In order to test the mediating effect, a Bootstrap procedure with repeated sampling of 1000 times was used. In this paper, 95% confidence interval of bias correction is calculated to test the significance of mediating effect. The effective sample size of this analysis is N = 579. Model fit was evaluated using multiple indices: (χ²/df) less than 5, RMSEA below 0.08, and CFI and TLI values exceeding 0.9 were considered indicative of good model fit.

## 5. Research findings

### 5.1. Reliability and validity tests

The reliability test of the Academic Pressure Scale for College Students demonstrated an internal consistency coefficient (Cronbach’s α = 0.917), indicating satisfactory reliability. KMO = 0.932, confirming the appropriateness of the data for factor analysis. Exploratory factor analysis revealed four underlying dimensions of academic pressure: personal, social, familial, and institutional pressure sources. The Bartlett’s test of sphericity yielded a statistically significant p-value (<0.0001), confirming substantial inter-item correlations and supporting construct validity.

For the Academic Procrastination Questionnaire for College Students, Cronbach’s α = 0.871, indicating adequate reliability. The KMO value (0.939) confirmed factor analysis suitability. Three dimensions of academic procrastination were identified: delayed initiation, poor completion, and inadequate planning. The Bartlett’s test (p < 0.0001) supported construct validity through significant inter-item correlations.

The Simplified Coping Style Questionnaire demonstrated, Cronbach’s α = 0.862, indicating acceptable reliability. With a KMO value of 0.886, factor analysis was deemed appropriate. Four coping dimensions were identified: self-adjustment, external support seeking, avoidance, and problem shelving. The Bartlett’s test (p < 0.0001) confirmed significant inter-item correlations and construct validity.

Collectively, all three scales exhibited α coefficients exceeding 0.85, indicating robust internal consistency. KMO values (>0.4) confirmed factor analysis suitability, while Bartlett’s tests (all p < 0.0001) supported construct validity (Table 2).

**Table 2:** Cronbach’s alpha values and Bart Sphericity Test.

Scales	Cronbach’s Alpha	KMO	Bart Sphericity Test (p)
Academic Stress	0.917	0.932	****
Academic Procrastination	0.871	0.939	****
Copeing Style	0.862	0.886	****

Note: **** = p < 0.001, *** = p < 0.001, ** = p < 0.01.

### 5.2. Common method bias test

Harman’s single-factor test was conducted via unrotated exploratory factor analysis of all measurement items. The results show that the explanation ratio of variance for the first common factor is 20.931%, which is less than 40%, and there is no serious common method bias problem.

### 5.3. Independent samples t-tests and pearson correlation analyses

Independent samples t-tests revealed significant differences (p < 0.05) in coping styles and academic procrastination scores between high and low academic pressure groups (Tables 3–5). Specifically, positive coping (p = 0.032), negative coping (p = 0.017), and academic procrastination (p < 0.001) scores differed significantly.

**Table 3:** Comparison of coping styles and academic procrastination scores of college students in high and low academic stress groups ($\bar{x}\pm s$ ).variables.

	high-pressure group	low pressure group	p	Lower 5%	Upper 5%
possitively response	35.388 ± 6.120	34.127 ± 7.182	0.046	−0.2024	2.724
negatively response	20.408 ± 4.887	17.578 ± 4.803	***	1.762	3.899
academic procrastination	63.864 ± 11.140	51.387 ± 11.248	***	10.005	14.948

Note: **** = p < 0.001, *** = p < 0.001, ** = p < 0.01.

**Table 4:** Comparison of the academic procrastination scores of high and low positive coping groups($\bar{x}\pm s$).

variable	high positive coping group	low positive coping group	p	Lower 5%	Upper 5%
academic procrstination	56.647 ± 12.380	55.622 ± 12.028	0.233	−3.78809	1.738

Note: **** = p < 0.001, *** = p < 0.001, ** = p < 0.01.

**Table 5:** Comparison of the academic procrastination scores of high and low negative coping groups ($\bar{x}\pm s$).

variable	high negative coping group	low negative coping group	p	Lower 5%	Upper 5%
academic procrstination	61.750 ± 12.307	53.339 ± 11.700	***	5.786	11.036

Note: **** = p < 0.001, *** = p < 0.001, ** = p < 0.01.

No significant differences (p > 0.05) in academic procrastination scores were observed between high and low positive coping groups (Table 4).

Similarly, academic procrastination scores did not differ significantly (p < 0.001) between high and low negative coping groups (Table 5).

Furthermore, Pearson correlation analyses demonstrated: (a) a weak positive correlation between academic pressure and negative coping (r = 0.288, p < 0.01); (b) a weak positive correlation between academic pressure and positive coping (r = 0.143, p < 0.01); (c) a moderate positive correlation between academic pressure and academic procrastination (r = 0.521, p < 0.01); (d) a low positive correlation between negative coping and academic procrastination (r = 0.318, p < 0.01); and (e) no significant correlation between positive coping and academic procrastination. These findings supported Hypotheses 1, 3, and 5 while rejecting Hypotheses 2 and 6 (Table 6).

**Table 6:** Correlation Coefficient of academic pressure, coping styles and academic procrastination (r).

Variables	M	SD	1	2	3	4
1.academic procrastination	57.571	11.275	1			
2.academic pressure	66.352	12.834	0.521***	1		
3.negative coping	18.216	4.755	0.318**	0.288**	1	
4.possitive coping	34.852	6.070	Insignificant	0.143**	0.252***	1

Note: **** = p < 0.001, *** = p < 0.001, ** = p < 0.01.

### 5.4. Stepwise regression analyses

Four stepwise regression models were constructed (Table 7). Model 1 demonstrated that academic pressure alone explained 27.1% of the variance in academic procrastination (β = 0.521, R² = 0.271, p < 0.001). Model 2 revealed that academic pressure explained 8.3% of the variance in negative coping (β = 0.288, R² = 0.083, p < 0.001), indicating poor fit. Model 3 showed that academic pressure explained 2.0% of the variance in positive coping (β = 0.143, R² = 0.020, p < 0.01), also indicating poor fit. Model 4 demonstrated that academic pressure and negative coping jointly explained 30.2% of the variance in academic procrastination (β₁ = 0.468, β₂ = 0.183, R² = 0.302, p < 0.001), outperforming the previous models.

**Table 7:** Model comparison.

Model	R^2^	p	VIF
Model1	0.271	0.000	1.000
Model2	0.083	0.000	1.000
Model3	0.020	0.001	1.000
Model4	0.302	0.000	1.000

Note: **** = p < 0.001, *** = p < 0.001, ** = p < 0.01.

Model 1 Predictors, Academic Stress. Dependent variable, Academic Procrastination.

Model 2 Predictors, Academic Stress. Dependent variable, Negative Coping style.

Model 3Predictors, Academic Stress. Dependent variable, Positive Coping style.

Model4 Predictors, Academic Stress, Negative Coping style. Dependent variable, Academic Procrastination.

### 5.5. Mediation effect tests

Model 4 was initially tested using Mplus, yielding χ²/df = 6.766, RMSEA = 0.100, CFI = 0.945, TLI = 0.917, and SRMR = 0.07. Although most indices indicated acceptable fit, the “inadequate planning” on “academic procrastination” factor loading (0.009, p = 0.451) was non-significant. After removing this factor, Model 4’ demonstrated improved fit (χ²/df = 4.828, RMSEA = 0.08, CFI = 0.97, TLI = 0.96, SRMR = 0.03), exceeding the criteria outlined in Section 3.4 (Table 8).

**Table 8:** Model Fit Information.

Model	χ^2^	df	RMSEA	CFI	TLI	SRMR
Model 4	162.395	24	0.100	0.945	0.917	0.000
Model 4’	82.074	17	0.081	0.973	0.956	0.030

Note: **** = p < 0.001, *** = p < 0.001, ** = p < 0.01.

Structural equation modeling revealed significant standardized paths (S2 Fig): academic pressure positively correlated with negative coping (β = 0.426, p < 0.001) and academic procrastination (β = 0.470, p < 0.001), while negative coping positively correlated with academic procrastination (β = 0.158, p < 0.05).

The Bootstrap procedure (1000 resamples) confirmed a significant mediating effect of negative coping (p = 0.029), accounting for 12.64% of the total effect (0.068). The direct effect of academic pressure on academic procrastination remained significant (p < 0.0001, 87.36% of total effect), supporting partial mediation (Hypothesis 7, Table 9).

**Table 9:** Value and significance test of mediation effect.

Path	Effect Size	S.E.	p
Indirect Effect (Academic Pressure-Negative Coping Style – Academic Procrastination)	0.068	0.036	0.029
Direct Effect (Academic Pressure – Academic Procrastination)	0.470	0.055	0.000
Total Effect = Indirect Effect+ Direct Effect	0.538	0.048	0.029

Note: **** = p < 0.001, *** = p < 0.001, ** = p < 0.01.

## 6. Discussion

### 6.1. Positive correlation between academic pressure and academic procrastination

It was initially found in this study that a significant positive correlation existed between academic pressure and an increase in academic procrastination (β = 0.470, p < 0.001). The Procrastination-Health Model has confirmed that procrastination, as a manifestation of self-regulatory failure, can lead to stress and subsequently impair physical and mental health. Subsequently, the question arises as to whether stress, in turn, leads to procrastination. Research on the academic procrastination and stress of nursing students in vocational colleges has revealed a correlation between academic procrastination and academic stress among nursing students [63]. Then, is a similar correlation observable among non-medical and non-nursing students? According to the ego depletion theory, sustained high levels of academic pressure can deplete students’ limited psychological resources and self-control abilities. Consequently, when faced with arduous or aversive learning tasks, their capacity to suppress procrastination impulses and adhere to goal-directed behaviors is reduced. Simultaneously, negative emotions such as anxiety and tension, which are associated with stress, can intensify individuals’ assessment of the negative valence of tasks. To seek immediate emotional relief, students are more likely to engage in alternative activities like entertainment, thereby delaying the initiation or completion of tasks. The findings of this study corroborate the vicious cycle of the pressure-procrastination bidirectional interaction, indicating that the highly competitive Chinese academic environment is associated with an increase in academic procrastination.

### 6.2. Mediating mechanism of negative coping styles

The most crucial finding of this study lies in the clarification of the partial mediating role of negative coping styles between academic pressure and academic procrastination. Path analysis revealed that academic pressure not only directly leads to procrastination but also indirectly exacerbates procrastination behavior by increasing students’ tendency to adopt negative coping styles, with the mediating effect accounting for 12.64%. This mechanism perfectly aligns with the core hypothesis of Sirois’s (2016) “Procrastination-Health” extended model [58]. This model posits that procrastinators tend to use emotion-focused avoidance strategies to cope with stress. Although these strategies temporarily alleviate negative emotions, they result in task accumulation and greater time pressure, thereby trapping individuals in a downward spiral of “pressure-avoidance-procrastination-greater pressure.” Our research successfully extends the explanatory power of this model from the general health domain to the academic context of Chinese university students, confirming that negative coping serves as a crucial psychological bridge between perceived stress and procrastination behavior.

### 6.3. Insignificant effect of positive coping styles

A weak positive correlation was found between academic pressure and positive coping styles (r = 0.143, p < 0.01), indicating that university students tend to initiate adaptive coping mechanisms under stressful conditions. However, the correlation between positive coping styles and academic procrastination was not significant. This result reveals a disconnect between coping styles and the effectiveness of behavioral regulation.

Firstly, the initiation of positive coping does not equate to its effective execution. According to the ego depletion theory, sustained pressure leads to psychological resource depletion, weakening individuals’ ability to implement self-regulatory strategies. Even if university students attempt to adopt positive coping styles such as problem-solving or seeking support, their limited executive function may hinder the formation of stable inhibition against procrastination behavior.

Secondly, structural academic pressure limits the effectiveness of positive coping. In the high-load, highly competitive environment of Chinese universities, individual efforts sometimes fail to receive timely positive feedback, easily triggering a sense of “ineffectiveness of effort.” When positive coping fails to significantly alleviate stress or improve task progress, its behavioral regulatory function is weakened, making it difficult to break the procrastination cycle.

Finally, there are limitations in the conceptual coverage of the measurement tool. The positive coping dimension in the Simplified Coping Style Questionnaire emphasizes general attitudes and fails to fully capture specific behavioral strategies with direct intervention effects on procrastination, such as “task decomposition” and “time monitoring,” potentially underestimating their actual regulatory role.

Therefore, although positive coping styles are cognitively activated, their behavioral transformation efficacy is insufficient under the dual influence of resource limitations and environmental constraints, leading to an insignificant correlation and mediating effect with academic procrastination. This result suggests that enhancing the practical effectiveness of positive coping requires a synergistic optimization of psychological resource support and structural interventions.

## 7. Theoretical and practical implications

Grounded in the extended “procrastination-health” model as a theoretical framework, this study employed empirical methods to investigate relationships among academic pressure, coping styles, and academic procrastination among university students. The derived conclusions bear substantial theoretical and practical significance. Theoretically, these findings refine the application of the “procrastination-health” model within Chinese university student populations, underscoring the heightened relevance of negative coping styles over positive coping styles in high-pressure cultural contexts. Practically, the results guide psychological intervention strategies in higher education: addressing academic procrastination requires not only skill-based interventions (e.g., time management training) but also systematic attention to pressure management and emotional regulation capacities. A critical intervention point involves assisting students in recognizing and modifying maladaptive avoidance responses to pressure, while fostering adaptive emotional regulation strategies to disrupt the “pressure → negative coping → procrastination” cycle and promote healthy academic behaviors. For individual students, these insights facilitate self-improvement through enhanced behavioral awareness—enabling retrospective analysis of pressure levels and coping strategy selection during procrastination episodes, thereby empowering intentional self-intervention, help-seeking, or strategy adaptation to achieve academic autonomy.

## 8. Limitations

Through empirical analysis, the partial mediating role of negative coping styles between academic stress and academic procrastination has been unveiled in this study. Nevertheless, certain limitations are still present in the research, as detailed in the following aspects.

Firstly, a cross-sectional data collection approach was adopted in this study. Such data are only capable of revealing the correlation and mediating relationships among variables, yet fail to clarify the causal relationships. Although the hypothesized path of “academic stress - coping styles - academic procrastination” has been validated by the structural equation model, there still exists a high probability of complex bidirectional or cyclic interaction relationships among academic stress, coping styles, and academic procrastination. It is recommended that future research employ a longitudinal tracking design or an experimental intervention design to more rigorously test the causal mechanisms among variables.

Secondly, all samples in this study were sourced from universities within a single region. The relatively small sample size and limited sampling scope may exert an adverse impact on the generalizability of the research conclusions. Given China’s vast territory, significant differences in economy, culture and education are observable across different regions. Moreover, universities at various levels also exhibit variations in terms of faculty strength, teaching resources, and training objectives. These factors are likely to result in systematic differences in the academic stress encountered by students and the coping resources accessible to them. Therefore, it is advisable for future research to conduct large-scale, multi-level random sampling across the country to assess the cross-group stability of the model.

In addition, self-report methods were predominantly utilized for data collection in this study. Although this method guarantees a certain level of efficiency in data collection, it may be susceptible to interference from various factors, such as social desirability bias and common method bias. Particularly in the measurement of procrastination behavior, self-assessment often lacks sufficient objectivity. Hence, it is suggested that future research combine multiple measurement methods, for instance, utilizing objective behavioral data like assignment submission times in the learning management system to quantify procrastination behavior, thereby enhancing the accuracy and reliability of the measurements.

Finally, this study centered on coping styles as the core mediating mechanism. However, a substantial amount of unexplained variance remains in the model, suggesting that numerous important factors have not been incorporated into the research scope. For instance, an individual’s emotion regulation ability, self-compassion, academic motivation and social support may all assume moderating or mediating roles in this mechanism. Consequently, it is recommended that future research models integrate these variables to construct a more comprehensive and in-depth explanatory framework for academic procrastination among Chinese college students, with the aim of more accurately uncovering the formation mechanisms and influencing factors of academic procrastination.

## 9. Conclusion

An empirical investigation was conducted in this study to examine the mechanisms underlying academic stress, coping styles, and academic procrastination within the Chinese cultural context. Findings confirm a statistically significant positive correlation between academic pressure and increased academic procrastination among university students. Crucially, the partial mediating role of negative coping styles in the relationship between academic pressure and procrastination was revealed. Specifically, academic stress was found to drive university students’ adoption of negative coping strategies—such as avoidance and self-blame—thereby intensifying procrastination behaviors. This discovery provides empirical validation for Sirois’s (2016) extended “procrastination-health” model within China’s high-pressure academic environment, delineating the causal pathway of “stress → negative coping → procrastination”. Notably, positive coping styles exhibited no significant effect, potentially attributable to the depletion of coping resources in China’s high-stress educational context, which may predispose students to select avoidance-oriented strategies.

## Supporting information

S1 Data Excel. (XLSX)

S2 Fig Structural Equation Model between Academic Pressure, Negative Coping Style and Academic Procrastination. (TIF)

S3 File Constructs and items. (PDF)
