# Supplementary figures and images for: Academic pressure and academic procrastination: The mediating role of negative coping strategies
Source: PLoS One. 2025 Dec 19;20(12):e0338956. doi: 10.1371/journal.pone.0338956 (PMC12716715; doi:10.1371/journal.pone.0338956)

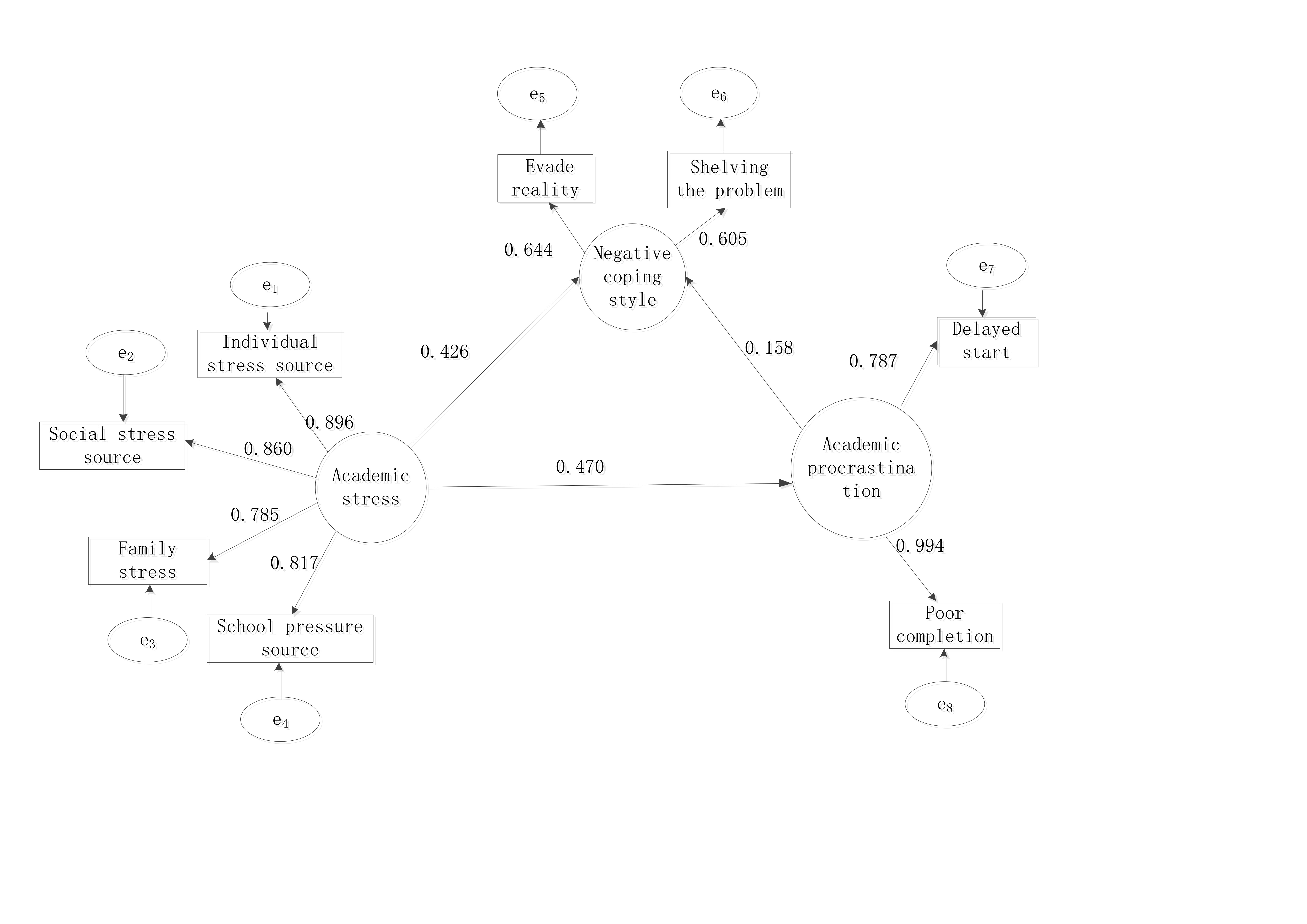

Supplement: S2 Fig — (TIF) [file pone.0338956.s002.tif]
