# Supplementary material for: Academic pressure and academic procrastination: The mediating role of negative coping strategies
Source: PLoS One. 2025 Dec 19;20(12):e0338956. doi: 10.1371/journal.pone.0338956 (PMC12716715; doi:10.1371/journal.pone.0338956)
Supplement: S3 File — (PDF) [file pone.0338956.s003.pdf]

Constructs and items

Dimension	Item	Resource
AP	(AP1) I often wait until just before class to rush to the classroom.<br>(AP2) I usually don’t put in effort and always cram for exams at the last minute.<br>(AP3) I can’t organize my schedule well.<br>(AP4) I failed to finish the books that I could have finished on time.<br>(AP5) I always rush to do my homework only when it’s about to be due.<br>(AP6) Sometimes, by the end of the day, I don't even know what I’ve done.<br>(AP7) When I study in my dormitory, I often stop to do other things.<br>(AP8) I make sure to organize my study materials so I can use them at any time.<br>(AP9) I rarely complete the tasks I set for myself on time.<br>(AP10) Before exams, I think about revising but never take any action.<br>(AP11) I never stick the study plans that I make for myself. | (AP12) For assignments or reports with deadlines, I procrastinate unless someone reminds me.<br>(AP13) I have a detailed plan for preparing for exams.<br>(AP14) I often miss opportunities because I didn’t take timely action.<br>(AP15) I often make excuses for not completing my academic tasks on time.<br>(AP16) I always postpone assignments or other academic tasks.<br>(AP17) I only start doing academic tasks when I can no longer delay them.<br>(AP18) I have a study plan every day.<br>(AP19) I often check the tasks I should complete before entertainment or going to bed.	Zhao (2007) College Students’ Academic Procrastination Questionnaire
AS	(AS1)I feel like there is not enough time, and thinking about it makes me anxious.<br>(AS2) There are too many graduates, and the job market is tough, making me feel a lot of academic pressure.<br>(AS3) My parents have very high expectations of my study.<br>(AS4) There are too many exams and certifications, and I feel stressed.	Liu (2015) College Students’ Academic Stress Scale

AS	(AS5) My academic workload is too heavy, and I feel pressured.<br>(AS6) Work now requires comprehensive skills, making my academic pressure greater.<br>(AS7) I hope to find a and earn money as soon as possible to support my parents.<br>(AS8) I study hard to get credits.<br>(AS9) My self-expectations are high, but the reality is quite different.<br>(AS10) I am worried about my ability to adapt to society in the future.<br>(AS11) My parents compare me to my peers which increases my pressure.<br>(AS12) The competition among classmates causes me stress.<br>(AS13) The unsuitable study environment makes me pressured.<br>(AS14) The pressure from exams and studies to enter national enterprises is overwhelming.<br>(AS15) My family's financial situation is tight, so I must study harder.<br>(AS16) I am not adapting well to the school environment.<br>(AS17) My learning methods are unscientific and my academic ability is limited.<br>(AS18) I am unsure about my future role in society.<br>(AS19) My parents hope I find a stable job soon.<br>(AS20) The pressure from romantic relationships has a significant impact on studying.	Liu (2015) College Students' Academic Stress Scale
CSS	(CSS1) I relieve stress through work, study, or other activities.<br>(CSS2) I talk to someone to vent my inner troubles.<br>(CSS3) I try to see the positive side of things.<br>(CSS4) I change my perspective and rediscover what is important in life.<br>(CSS5) I don't take problems too seriously.<br>(CSS6) I stick to my position and fight for what I want.<br>(CSS7) I come with different solutions to problems.<br>(CSS8) I seek advice from relatives, friends, classmates.<br>(CSS9) I change my methods or resolve personal issues.<br>(CSS10) I learn from how others handle similar difficulties.<br>(CSS11) I engage in hobbies or participate in cultural and sports activities.<br>(CSS12) I try to suppress feelings of disappointment, regret, sadness, or anger.	Xie (1998) Simplified Coping Style Questionnaire

CSS	(CSS13) I try to take a break or vacation, temporarily putting the problem aside.<br>(CSS14) I relieve stress by smoking, drinking, or eating.<br>(CSS15) I believe time will change the situation, and the only thing I can do is wait.<br>(CSS16) I try to forget the whole situation.<br>(CSS17) I rely on others to solve my problems.<br>(CSS18) I accept the reality because there is no other choice.<br>(CSS19) I fantasize that a miracle will change the situation.<br>(CSS20) I comfort myself.	Xie (1998) Simplified Coping Style Questionnaire
